# Supplementary material for: Accounting for small variations in the tracrRNA sequence improves sgRNA activity predictions for CRISPR screening
Source: Nat Commun. 2022 Sep 6;13:5255. doi: 10.1038/s41467-022-33024-2 (PMC9448816; doi:10.1038/s41467-022-33024-2)
Supplement: Supplementary file 1 — Supplementary Information [file 41467_2022_33024_MOESM1_ESM.pdf]

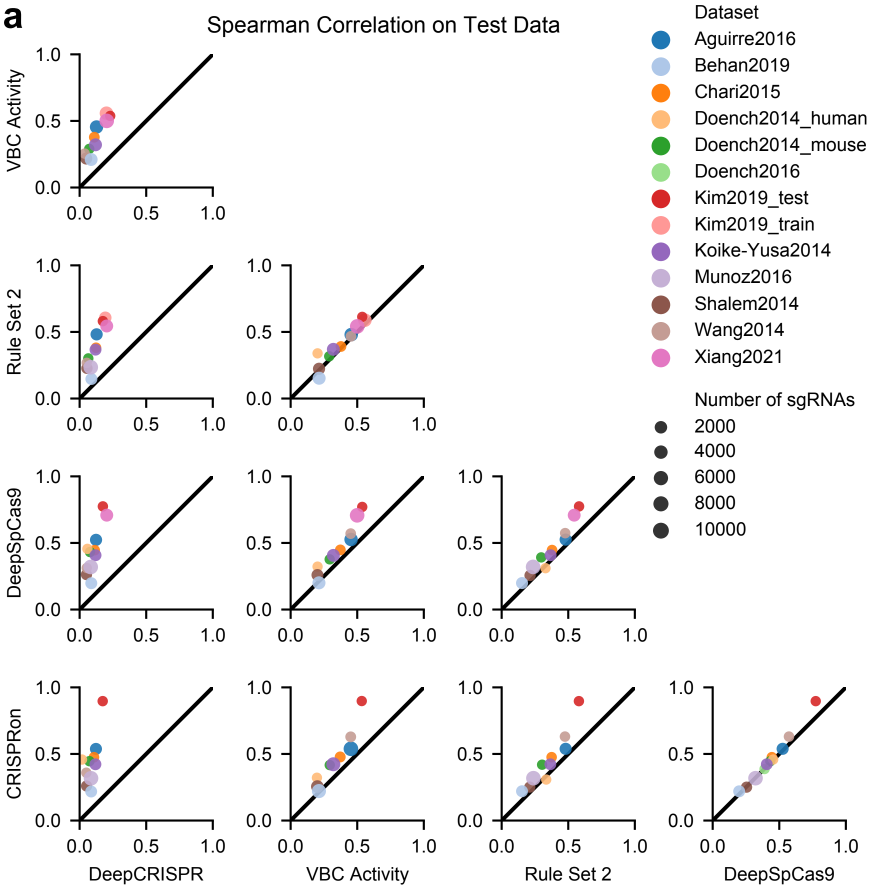

Supplementary Figure 1: Development of Rule Set 3 (Sequence). a) Spearman correlations between observed and predicted activity for the collated datasets across existing models. The number of sgRNAs used to calculate Spearman correlations for a given dataset differs between scatter plots and are indicated by point size. b) Pearson correlations on held out folds between the predicted and observed activity differences between sgRNAs in the Behan and Aguirre datasets (CV = cross validation; n test sgRNAs per fold=175 or 176). c) Spearman correlations between observed and predicted activity for six held out datasets across previous models and Rule Set 3 (Sequence). Points represent the observed spearman correlation and error bars represent 95% bootstrapped confidence intervals (n=1000 resamplings; high=97.5th percentile, low=2.5th percentile). d) Comparison of Rule Set 2 and Rule Set 3 (Sequence) on held out test sgRNAs with Hsu tracrRNA (n=25,268).

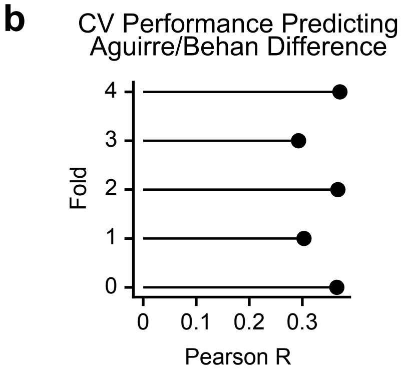

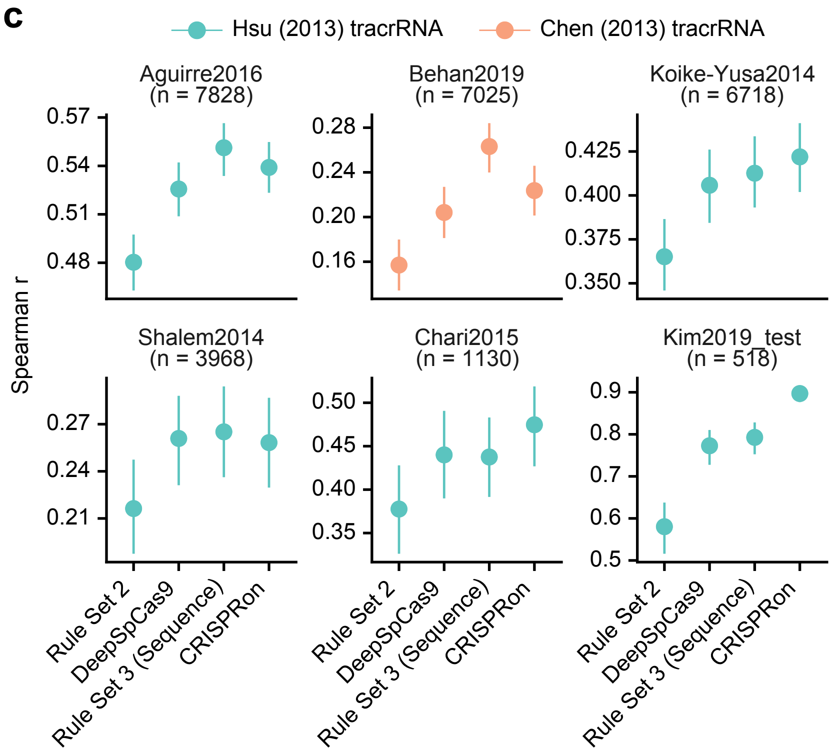

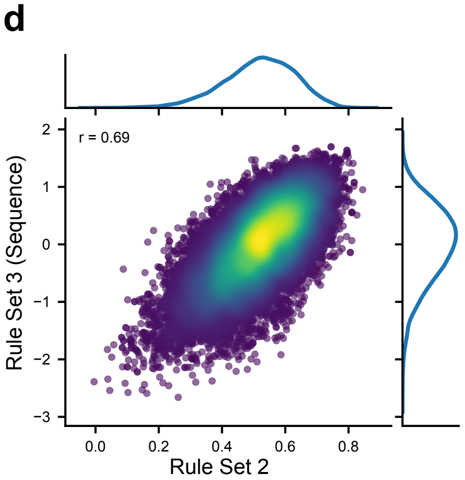

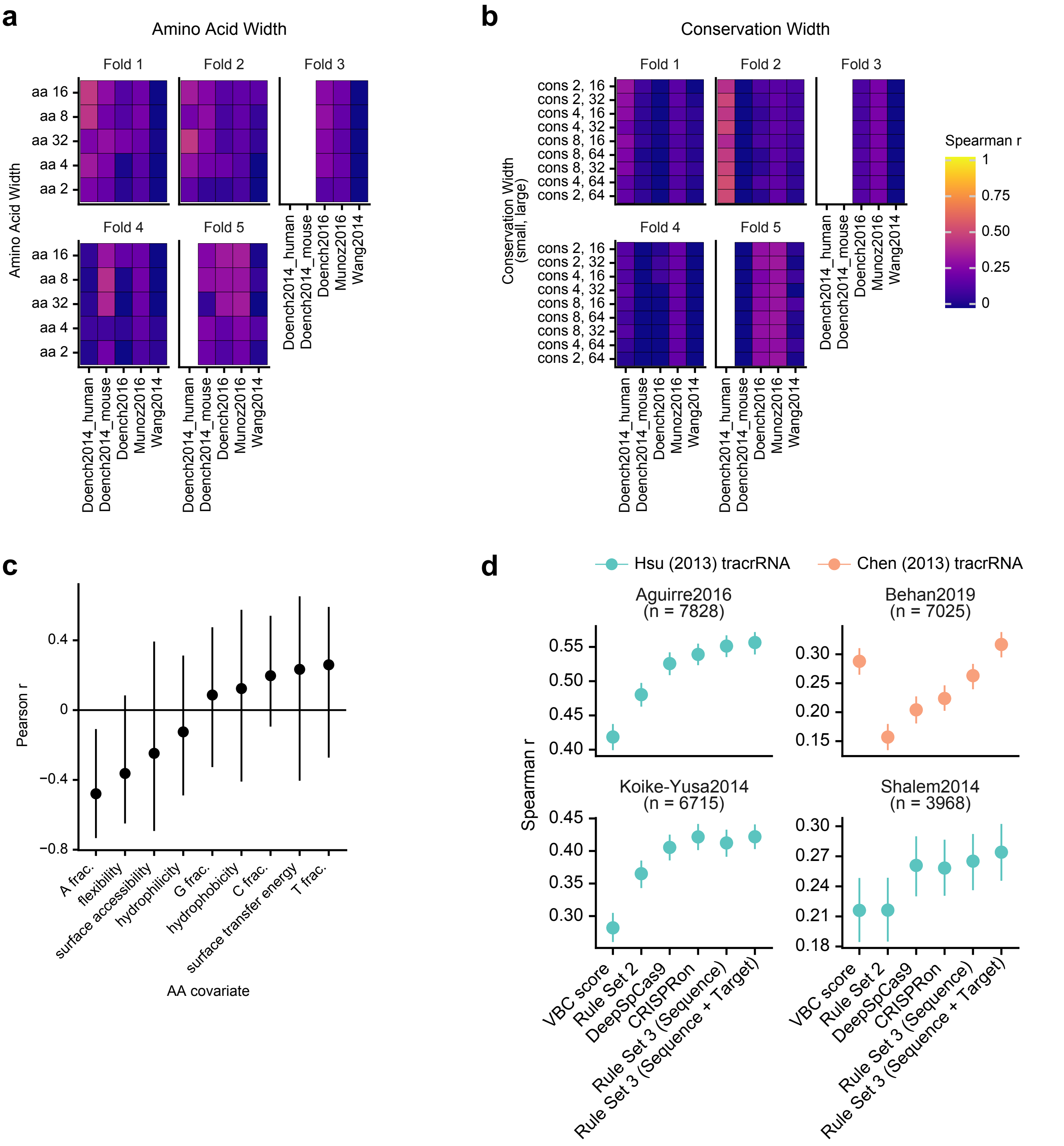

Supplementary Figure 2: Development of Rule Set 3 (Target). a) Spearman correlations between amino acid window widths around the cut site and cross-validation held out set sgRNA activity. For each fold all sgRNAs targeting a gene were assigned to either the training or testing set and not both. Due to the small number of genes in the Doench 2014 datasets, some folds do not have test sgRNAs from these datasets. b) Spearman correlations between conservation widths around the cut site and cross-validation held out set sgRNA activity. c) Pearson correlation between amino acid biochemical properties and the directional importance of amino acids in the target model (n=26). Directional importance was calculated by taking the mean absolute shap value of an amino acid feature and multiplying by the correlation direction of the shap values and observed activity. Points represent the median pearson correlation over 1,000 bootstrap resamplings and error bars represent 95% confidence intervals (high=97.5th percentile, low=2.5th percentile) d) Spearman correlations between observed and predicted activity for four held out datasets across previous models and Rule Set 3 models. Points represent the observed spearman correlation and error bars represent 95% bootstrapped confidence intervals (n=1000 resamplings; high=97.5th percentile, low=2.5th percentile).

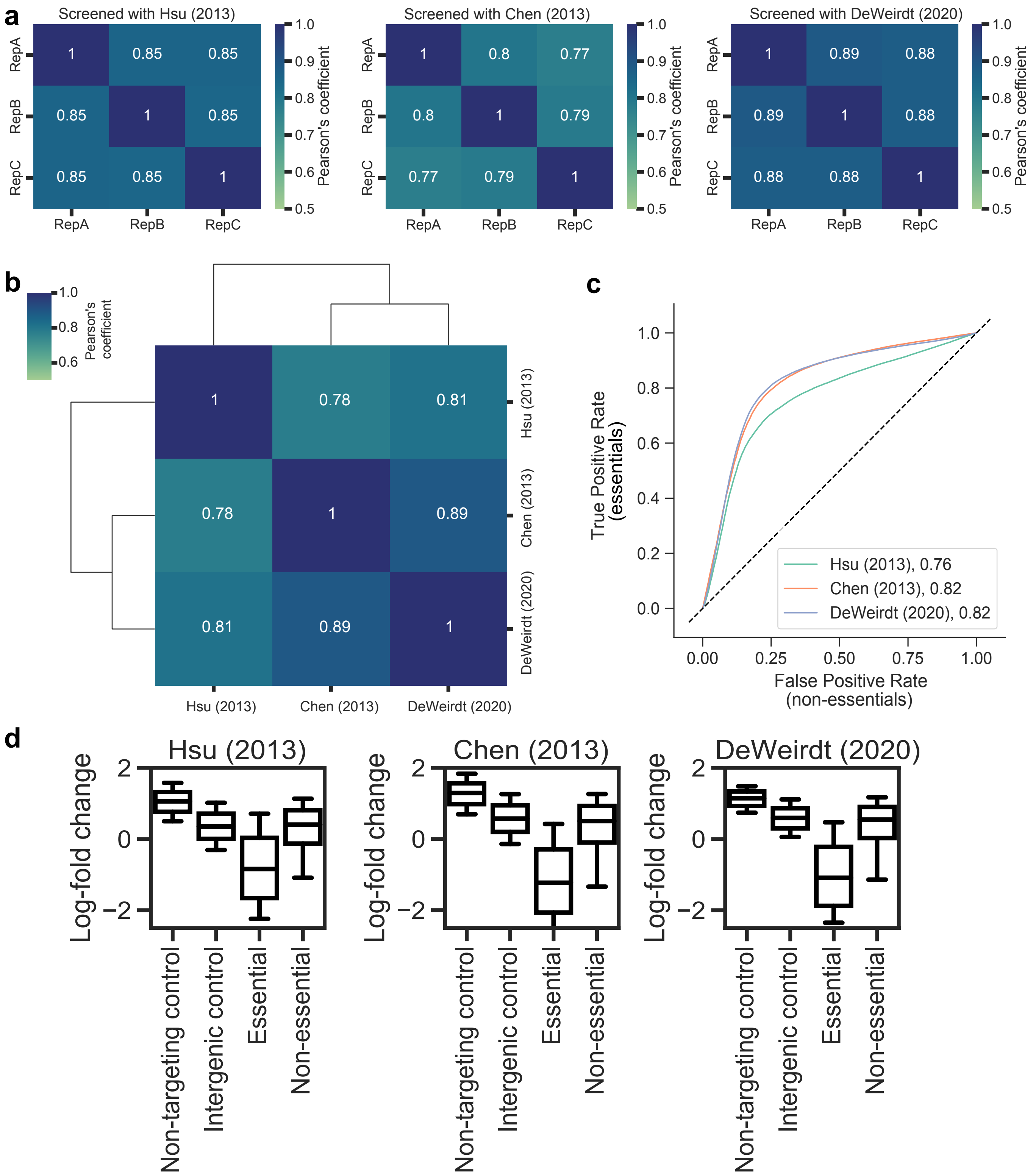

Supplementary Figure 3: Essential/non-essential tiling library screened with tracrRNA variants. a) Replicate correlation (Pearson's r) for the essential/non-essential library screened with the three tracrRNA variants in triplicate. b) Correlation (Pearson's r) between the average log-fold changes for the essential/non-essential library across the three screens. c) ROC plots for the essential/non-essential screen performed with each tracrRNA variant, using sgRNAs targeting essential genes as positive controls and sgRNAs targeting non-essential genes as negative controls. AUC is reported in the graph legend. x=y line is shown. d) Log-fold changes for the different spacer categories in each of the three essential/non-essential screens. Boxes show 25th and 75th percentiles as minima and maxima and the center represents the median; whiskers show 10th and 90th percentile. Number of spacers in each category are as follows: Non-targeting control: 998, Intergenic control: 1000, Essential: 48730, Non-essential: 33621.
