# Supplementary material for: Accounting for small variations in the tracrRNA sequence improves sgRNA activity predictions for CRISPR screening
Source: Nat Commun. 2022 Sep 6;13:5255. doi: 10.1038/s41467-022-33024-2 (PMC9448816; doi:10.1038/s41467-022-33024-2)
Supplement: Supplementary file 2 — Reporting Summary [file 41467_2022_33024_MOESM2_ESM.pdf]

nature portfolio

Corresponding author(s): John Doench

Last updated by author(s): Aug 10, 2022

Reporting Summary

Nature Portfolio wishes to improve the reproducibility of the work that we publish. This form provides structure for consistency and transparency in reporting. For further information on Nature Portfolio policies, see our Editorial Policies and the Editorial Policy Checklist.

Statistics

For all statistical analyses, confirm that the following items are present in the figure legend, table legend, main text, or Methods section.

n/a	Confirmed	
☐	☒	The exact sample size (n) for each experimental group/condition, given as a discrete number and unit of measurement
☐	☒	A statement on whether measurements were taken from distinct samples or whether the same sample was measured repeatedly
☐	☒	The statistical test(s) used AND whether they are one- or two-sided<br>*Only common tests should be described solely by name; describe more complex techniques in the Methods section.*
☒	☐	A description of all covariates tested
☒	☐	A description of any assumptions or corrections, such as tests of normality and adjustment for multiple comparisons
☐	☒	A full description of the statistical parameters including central tendency (e.g. means) or other basic estimates (e.g. regression coefficient) AND variation (e.g. standard deviation) or associated estimates of uncertainty (e.g. confidence intervals)
☐	☒	For null hypothesis testing, the test statistic (e.g. F, t, r) with confidence intervals, effect sizes, degrees of freedom and P value noted<br>*Give P values as exact values whenever suitable.*
☒	☐	For Bayesian analysis, information on the choice of priors and Markov chain Monte Carlo settings
☒	☐	For hierarchical and complex designs, identification of the appropriate level for tests and full reporting of outcomes
☐	☒	Estimates of effect sizes (e.g. Cohen's d, Pearson's r), indicating how they were calculated

Our web collection on statistics for biologists contains articles on many of the points above.

Software and code

Policy information about availability of computer code

Data collection

PoolQ was used to deconvolute screens, which is openly available at https://portals.broadinstitute.org/gpp/public/software/poolq

CRISPick has been updated based on the results of this study and is available at broad.io/crispick

To build the target model, we used Ensembl's REST API to query the amino acid sequence around the cut site of each sgRNA (accessed August 9, 2021). We used biopython to get biochemical properties of these amino acid sequences (version 1.79). Ensembl's REST API was also used to obtain protein domain features. We used the UCSC genome browser's REST API to get PhyloP conservation scores for each sgRNA (accessed August 9, 2021).

Data analysis

Screens were analyzed using custom code written in R version 3.5.1 or Python3 that is available on GitHub as indicated in the manuscript. Python3 and PRISM Graphpad (v9) were used for visualization.

To build Rule Set 3 (Sequence) sgRNA features were extracted using the custom Python package sglearn (version 1.2.3; https://github.com/gpp-rnd/sglearn).

To fit an optimal gradient boosting model from sequence features, we used the gradient boosting framework from LightGBM (version 3.2.0)30 and tuned hyperparameters using Tree Structured Parzen Estimators from Optuna (version 2.7.0). We tuned the number of leaves (between 8 and 256) and minimum number of samples in a child (between 8 and 256) over 50 hyperparameter iterations. We fixed the learning rate to be 0.01 and used 5,000 boosted trees. All other parameters were kept default. To evaluate each set of hyperparameters we split our dataset into five folds using the StratifiedGroupKFold splitter from scikit-learn. Feature importances were calculated using the shap package in Python (version 0.39).

All custom code used for analysis and example notebooks are available on GitHub: https://github.com/broadinstitute/rs3_manuscript

Code for developing the on-target model can be found on GitHub: https://github.com/gpp-rnd/rs_dev

A python package for scoring sgRNA sequences with Rule Set 3 can be found on GitHub: https://github.com/gpp-rnd/rs3

For manuscripts utilizing custom algorithms or software that are central to the research but not yet described in published literature, software must be made available to editors and reviewers. We strongly encourage code deposition in a community repository (e.g. GitHub). See the Nature Portfolio guidelines for submitting code & software for further information.

Data

Policy information about availability of data

All manuscripts must include a data availability statement. This statement should provide the following information, where applicable:

- Accession codes, unique identifiers, or web links for publicly available datasets
- A description of any restrictions on data availability
- For clinical datasets or third party data, please ensure that the statement adheres to our policy

The manuscript includes a data availability statement, as also indicated in the editorial checklist, and pasted here as well:
The read counts for all screening data generated in this study have been deposited in the Sequence Read Archive database under accession code PRJNA832308. The subsequent analyses data generated in this study are provided in the Supplementary Data/Source Data file. Source data are provided with this paper.

Field-specific reporting

Please select the one below that is the best fit for your research. If you are not sure, read the appropriate sections before making your selection.

☒ Life sciences ☐ Behavioural & social sciences ☐ Ecological, evolutionary & environmental sciences

For a reference copy of the document with all sections, see nature.com/documents/nr-reporting-summary-flat.pdf

Life sciences study design

All studies must disclose on these points even when the disclosure is negative.

Sample size	All screens were performed such that each guide was present, on average, in at least 500 cells. This sample size was determined by counting the number of cells in the population and dividing by the number of guides in the library.
Data exclusions	no data were excluded
Replication	All screens were performed in triplicate, successfully.
Randomization	No randomization was necessary for the experimental design. The randomization of training the ML model is provided in the methods section, and pasted here as well:<br>We split the data such that all sgRNAs targeting a gene were either in the train set or the test set for each fold. We also tried to represent each dataset source (e.g. Doench 2016 sgRNAs) proportionally in all of the folds, such that each test set had some sgRNAs from each source. We found that a model with a maximum of 111 leaves per base estimator and a minimum of 199 samples per child performed best. We used these hyperparameters to train our final model.
Blinding	No blinding was necessary, as we have no idea how that would be relevant for this study, as no subjective assessments were required.

Reporting for specific materials, systems and methods

We require information from authors about some types of materials, experimental systems and methods used in many studies. Here, indicate whether each material, system or method listed is relevant to your study. If you are not sure if a list item applies to your research, read the appropriate section before selecting a response.

Materials & experimental systems

n/a	Involved in the study
x	Antibodies
	x Eukaryotic cell lines
x	Palaeontology and archaeology
x	Animals and other organisms
x	Human research participants
x	Clinical data
x	Dual use research of concern

Methods

n/a	Involved in the study
x	ChIP-seq
x	Flow cytometry
x	MRI-based neuroimaging

Eukaryotic cell lines

Policy information about cell lines

Cell line source(s)	A375 cells were acquired from the Cancer Cell Line Encyclopedia.<br>HEK293T - ATCC (CRL-3216)
Authentication	Cell lines were authenticated by SNP profiling upon acquisition
Mycoplasma contamination	Cell lines were routinely tested for mycoplasma (~monthly) and were negative.
Commonly misidentified lines<br>(See ICLAC register)	None.
