# Supplementary material for: Accounting for small variations in the tracrRNA sequence improves sgRNA activity predictions for CRISPR screening
Source: Nat Commun. 2022 Sep 6;13:5255. doi: 10.1038/s41467-022-33024-2 (PMC9448816; doi:10.1038/s41467-022-33024-2)
Supplement: Supplementary file 4 — Description of Additional Supplementary Files [file 41467_2022_33024_MOESM4_ESM.pdf]

Title: Supplementary Data 1:
Description: On-target dataset summary. Associated with Fig. 1.

Title: Supplementary Data 2:
Description: Training and testing data for Rule Set 3 (Sequence). Associated with Fig 1.

Title: Supplementary Data 3:
Description: Feature names and descriptions. Associated with Fig. 1.

Title: Supplementary Data 4:
Description: Essential/non-essential read counts, library annotation. Associated with Fig 2.

Title: Supplementary Data 5:
Description: On target model Spearman correlations, Rule Set 3 scores for tracrRNA variants, LFCs for Rule Set 2 and Rule Set 3 guide picking, SSMD scores. Associated with Fig 2.

Title: Supplementary Data 6:
Description: z-score log-fold changes and G/T spacer abundances. Associated with Fig 3.
