# Supplementary material for: Accounting for small variations in the tracrRNA sequence improves sgRNA activity predictions for CRISPR screening
Source: Nat Commun. 2022 Sep 6;13:5255. doi: 10.1038/s41467-022-33024-2 (PMC9448816; doi:10.1038/s41467-022-33024-2)
Supplement: Supplementary file 3 — Peer Review File [file 41467_2022_33024_MOESM3_ESM.pdf]

Peer Review File

Accounting for small variations in the tracrRNA sequence improves sgRNA activity predictions for CRISPR screening

Open Access This file is licensed under a Creative Commons Attribution 4.0 International License, which permits use, sharing, adaptation, distribution and reproduction in any medium or format, as long as you give appropriate credit to the original author(s) and the source, provide a link to the Creative Commons license, and indicate if changes were made. In the cases where the authors are anonymous, such as is the case for the reports of anonymous peer reviewers, author attribution should be to 'Anonymous Referee' followed by a clear attribution to the source work. The images or other third party material in this file are included in the article's Creative Commons license, unless indicated otherwise in a credit line to the material. If material is not included in the article's Creative Commons license and your intended use is not permitted by statutory regulation or exceeds the permitted use, you will need to obtain permission directly from the copyright holder. To view a copy of this license, visit http://creativecommons.org/licenses/by/4.0/.

Editorial Note: This manuscript has been previously reviewed at another journal that is not operating a transparent peer review scheme. This document only contains reviewer comments and rebuttal letters for versions considered at *Nature Communications*. Mentions of prior referee reports have been redacted.

Reviewers' Comments:

Reviewer #1:
Remarks to the Author:
I was asked to comment not only on whether the points I had raised previously were addressed in the revised version, but also whether the points raised by Referee 3 were addressed.
Referee 1's points: All points have been adequately addressed.
Referee 3's points: Referee 3 mainly asked for clarifications and additional explanations, which the authors have now provided. Several of the many points raised seem inconsequential, and the review overall contains little constructive criticism that could be addressed to improve the manuscript. Where this was possible, the authors have done so in the revised version.
Thus, the manuscript is in my opinion now suitable for publication in Nature Communications.
